# Unveiling dental diagnostic dilemmas: a national survey of US dentists

**DOI:** 10.1186/s12903-025-07531-9

**Published:** 2025-12-23

**Authors:** Enihomo Obadan-Udoh, Rachel Howard

**Affiliations:** 1 Division of Population Health, University of Utah School of Dentistry, 530 Wakara Way, Salt Lake City, UT 84108 USA; 2 Department of Preventive and Restorative Dental Sciences, University of California San Francisco School of Dentistry, CA 94143 San Francisco, US

**Keywords:** Diagnostic errors, Dental care, General dentists, Specialist dentists, Patient safety

## Abstract

**Background:**

In dentistry, very little is known about diagnostic errors (DEs) despite their critical role in assessing patient safety. Many DE cases significantly impact the patient’s quality of life and daily function without necessarily causing medical harm. The primary goal of this study was to describe dentists past experiences or observations of DEs and their associated characteristics.

**Methods:**

We conducted a national cross-sectional study of US dentists (May-June, 2021). The primary outcomes were self-reported personal experiences of DEs and observations of DEs made by other dentists. Secondary outcomes included: dental conditions frequently associated with DEs, phase of care, contributory factors, and potential interventions. We also explored the associations between provider demographics and practice characteristics with the primary outcomes.

**Results:**

627 total responses were received from participants. About 40% of dentists reported observing a DE made by another dentist on a daily or weekly basis, while only 12.4% admitted to personally making a DE. The dental conditions most frequently selected by dentists as associated with DEs included: diseases of pulp, periapical tissues, and other disorders of the teeth and supporting structures (45%), acute and chronic sinusitis (44.6%), and head and neck cancers/neoplasms (43.9%). Younger dentists and those who attended to 61 + patients/week were significantly more likely to report personally making DEs.

**Conclusion:**

US Dentists report personally making or observing others making DEs frequently. New, innovative strategies are needed to reduce or eliminate the occurrence of DEs.

**Supplementary Information:**

The online version contains supplementary material available at 10.1186/s12903-025-07531-9.

## Background

A diagnostic error (DE) is defined as “a diagnosis that was unintentionally delayed (sufficient information was available earlier), wrong (another diagnosis was made before the correct one), or missed (no diagnosis was ever made), as judged from the eventual appreciation of more definitive information” [1]. A diagnosis in dentistry is the establishment of a disease or condition based on a patient’s clinical history, examination, testing, and radiographic findings. In dentistry, very little is known about diagnostic errors despite their critical role in assessing patient safety [2–10]. A recent study of diagnostic errors in periodontal diseases found that about one-third of the cases were misclassified [11]. Interviews with dental faculty members in another study revealed the prevailing belief that DEs only occurred when a provider’s treatment resulted in harm; this excluded delayed or missed diagnoses [12]. This belief aligns with the “misdiagnosis-related harm definition” posited by Newman-Toker et al. [13–16] However, DEs can significantly impact a patient’s quality of life and daily function even when they do not cause severe medical harm [9–11]. Other definitions of DEs emphasize the importance of communicating the diagnosis to the patient and the need to identify a missed opportunity in the diagnostic process [17–19]. 

A recent review of dental malpractice claims revealed that 8.7% of all paid dental claims over a 30 -year period were related to DEs, with majority (78.6%) being classified as “missed diagnoses” [20]. Dental patient interviews revealed that patients often endured prolonged suffering, unnecessary treatment, altered care-seeking behaviors, and financial difficulty, and became less trusting of dentists when they experienced a DE [21]. A scoping review of dental adverse events also revealed that 23% were associated with a delay in appropriate treatment, disease progression, or unnecessary interventions resulting from misdiagnoses [22]. These studies highlight the importance of understanding the mechanisms for DE occurrences in dentistry.

Understanding DEs in dentistry is further obscured by the lack of widespread adoption of standardized dental diagnostic codes [23], poor documentation in electronic dental records [24], and dependence on diagnostic tools with limited inter-rater reliability, such as clinical and radiographic observations [25–28]. Traditional approaches, such as chart audits, that have been utilized in medicine to understand DEs, are less effective in dentistry for these reasons [29–31]. Autopsies, also have limited utility in general dentistry, especially when the source of diagnosis (e.g. the tooth), has been removed or transformed during treatment. Most dental providers infer the initial dental diagnosis based on the treatment provided, patient descriptions of prior symptoms/treatment, or by examining prior treatment records.

To deepen our understanding of dental diagnostic errors, we conducted a national survey of dentists in the United States (US). The primary goal of this study was to describe dentists’ past experiences or observations of DEs and their associated characteristics.

## Methods

### Study design

We conducted a national cross-sectional study of dentists in the US. The study was deemed exempt by the Human Subjects Protection Program at the University of California San Francisco (#20-31017). Informed consent was obtained when participants read the information sheet and clicked on the link to complete the survey.

### Study population

A sample of 40,000 dentists was created through a random selection from the American Dental Association’s (ADA) Masterfile [32] of dentists (practicing and nonpracticing) in the United States that met the inclusion criteria. The inclusion criteria were: the possession of an active dental license in the two years preceding the study (January 2018-December 2020), and an email address. The sample included dentists stratified by their demographic characteristics (age, year of graduation, sex), contact information (email addresses), specialty, and census region. There was no significant difference between the distribution of the dentists in the study sample and the eligible dentists in the Masterfile (see supplement).

### Study procedures

Participants were invited to complete an electronic survey in RedCap via email which contained a study information sheet (see supplement). Informed consent was obtained by clicking on the survey link. To minimize email fatigue, researchers sent three email reminders over the one-month study period (May 24 – June 25, 2021) as recommended by the ADA Health Policy Institute. Participants were incentivized to complete the survey by enrolling them in a raffle draw with a chance to win one of ten $100 gift cards. The estimated sample size needed was 384 (significance level, a = 0.05; confidence interval = 0.5; total US dentist population (2021) = 202,304) based on the estimated prevalence (*p* = 50%) of self-reported experiences of DEs among dentists. (Note: A response rate of < 5% was typical for ADA surveys of dentists in 2021 according to the ADA Health Policy Institute. Due to the sensitive nature of the topic, a lower response rate was anticipated, therefore, email invitations were sent to all 40,000 dentists).

### Study instrument

A self-administered 20-item questionnaire was developed by the research team, leveraging questions from analogous studies conducted by Schiff et al. and Perrem et al. [33, 34] These questions were subsequently tailored to align with dental terminology. Face and content validity assessment was conducted with a cohort of ten dentists. Dentists evaluated each question using a Likert scale, assessed its relevance and ease of comprehension, and offered suggestions for modifications. The final survey instrument was developed after incorporating the feedback from the validity study. It comprised three distinct sections (See supplement):

- Section 1: Professional Opinion About Diagnostic Errors in Dentistry
- Section 2: Personal Experience with Diagnostic Errors
- Section 3: Provider and Practice Characteristics

Participants were provided with a DE definition sourced from Graber et al. [1] According to this framework, a diagnostic error is described as “a diagnosis that was either unintentionally delayed (i.e. sufficient information was available earlier), or wrong (i.e. another diagnosis was made before the correct one), or missed (i.e. no diagnosis was ever made), as judged from the eventual appreciation of more definitive information.” [1] This definition was selected because of its ease of operationalization and direct clinical relevance.

### Measures

The study focused on describing primary and secondary outcome measures pertaining to dental DEs. Primary outcome measures encompassed self-reported experiences of DEs and observations of DEs made by other dental providers. These were categorized into their frequency ranging from “Never” to “Daily” for the descriptive analysis, and “more frequent” (daily to quarterly) to “less frequent” (yearly to never), for the regression models. Secondary outcomes included: dental conditions commonly associated with DEs (organized into 16 broad disease categories), the phase of care most associated with DEs (categorized using the Diagnostic Error Evaluation and Research (DEER) taxonomy) [34, 35], factors contributing to DEs (categorized into cognitive, system-related, and situational factors) [1, 33], and interventions recommended to mitigate their occurrence (categorized into clinician-focused and system-focused interventions) [1, 33]. The Diagnostic Error Evaluation and Research (DEER) taxonomy, as established by Schiff [34, 35], defines seven crucial stages in the diagnostic process, each harboring the potential for the occurrence of a diagnostic error. These stages include “access/presentation, history, physical exam, tests, assessment, referral/consultation, and monitoring/follow-up” [34, 35]. Provider demographics (age, sex, specialty (general dentist vs. specialist dentist), years in practice (Less than or equal to 10 years vs. greater than 10 years), formal DE training (predoctoral, postdoctoral, or both), and practice characteristics (patient volume, primary practice setting, geographic location (US census division)) were also assessed.

### Data analysis

Descriptive statistics were performed for all outcome measures and explanatory variables to provide a comprehensive overview of DEs in dentistry. Chi-Square ($\:\chi\:$^2^) test and multivariable logistic regression (generalized linear models with Poisson distribution) were used to assess associations (incidence rate ratios) between the explanatory variables and the outcome measures. Responses with missing data points were excluded from the analyses for each variable (Listwise deletions). All tests were performed using STATA 16^Ⓡ^ .

## Results

### Description of provider and practice characteristics

627 total responses were received (1.6% response rate) at the end of the study period. After data cleaning and validation, 462 participant responses were retained for data analysis. Complete responses were only available in 334 (72.3%) of cases. The total number of available responses for each variable is shown in Tables 1, 2, 3 and 4.

Majority of the participants were aged 65–74 years (24.3%), male (68.4%), non-Hispanic White (80.9%), general dentists (69.5%), and had spent > 25 years in practice (50.3%). Pediatric dentists (6.6%), endodontists (5.1%), and oral and maxillofacial surgeons (5.1%) had the highest participation rates from specialists, while there were no responses from specialists in oral pathology and dental anesthesiology. The states with the highest number of participants were California (18.6%), New York (8.1%), and Texas (2.7%). Although there was representation from all geographic census divisions in the US, there were no responses received from dentists practicing in eight states (Arkansas, Delaware, Hawaii, Mississippi, Montana, Rhode Island, South Dakota, and Wyoming). No additional efforts (beyond the email invitations and reminders) were made to obtain responses from these unrepresented states. Majority of the participants practiced in small private practices (i.e. solo, small group containing 2–9 dentists; 67.4%), saw > 40 patients per week (59.8%), and had received formal training on DEs either at the predoctoral (37.7%) or postdoctoral (31.7%) level or both. Almost half (45.5%) reported receiving no formal training on DEs (Table 1).

**Table 1:** Provider and practice characteristics of respondents (total, *n* = 334)

	(*n*)	(%)
Gender		
Female	103	30.8
Male	228	68.3
Prefer Not to Answer	3	0.9
Age		
18–24 years	1	0.3
25–34 years	44	13.2
35–44 years	69	20.7
45–54 years	62	18.6
55–64 years	67	20.1
65–74 years	81	24.3
75–84 years	10	3.0
Race		
Hispanic or Latino	30	9.0
Black or African American	5	1.6
Middle Eastern or North African	7	2.3
AI/AN/NH/OPI	2	0.7
Asian	34	11.2
White	246	80.9
Two or more races	2	0.7
Other races	8	2.6
Specialty		
General Dentistry	232	69.5
Dental Anesthesiology	0	0
Dental Public Health	7	2.1
Endodontics	17	5.1
Prosthodontics	10	3.0
Pediatric Dentistry	22	6.6
Periodontics	12	3.6
Oral and Maxillofacial Surgery	17	5.1
Oral Pathology	0	0
Oral Medicine	1	0.3
Oral and Maxillofacial Radiology	1	0.3
Orthodontics	8	2.4
Orofacial Pain	7	2.1
Years in Practice		
0–5 years	37	11.1
6–10 years	38	11.4
11–15 years	30	9.0
16–20 years	28	8.4
21–25 years	33	9.9
26 years or more	168	50.3
US Census Division		
New England	17	5.1
Middle Atlantic	46	13.8
East North Central	53	15.9
West North Central	31	9.3
South Atlantic	48	14.4
East South Central	9	2.7
West South Central	34	10.2
Mountain	28	8.4
Pacific	67	20.1
Unknown	1	0.0
Primary Practice Setting		
Academic Dental Center	33	9.9
Community-Based Dental Center	17	5.1
Small Private Practice	225	67.4
Large Private Practice	23	6.9
Hospital	15	4.5
Military	7	2.1
Other	14	4.2
Approximate Number of Patients Seen Weekly		
1–20 patients	55	16.5
21–40 patients	78	23.4
41–60 patients	102	30.5
61 patients or more	98	29.3
Not Applicable	1	0.3
Diagnostic Error Training Received^§^		
At the predoctoral level	126	37.7
At the postdoctoral level	106	31.7
None	152	45.5
TOTAL	334	100%

^§^Select all that apply

^*^AI= American Indian; AN= Alaskan Native; NH: Native Hawaiian; OPI: Other Pacific Islander

### Diagnostic errors by Experienced/Observed by dentists

**Fig. 1:**
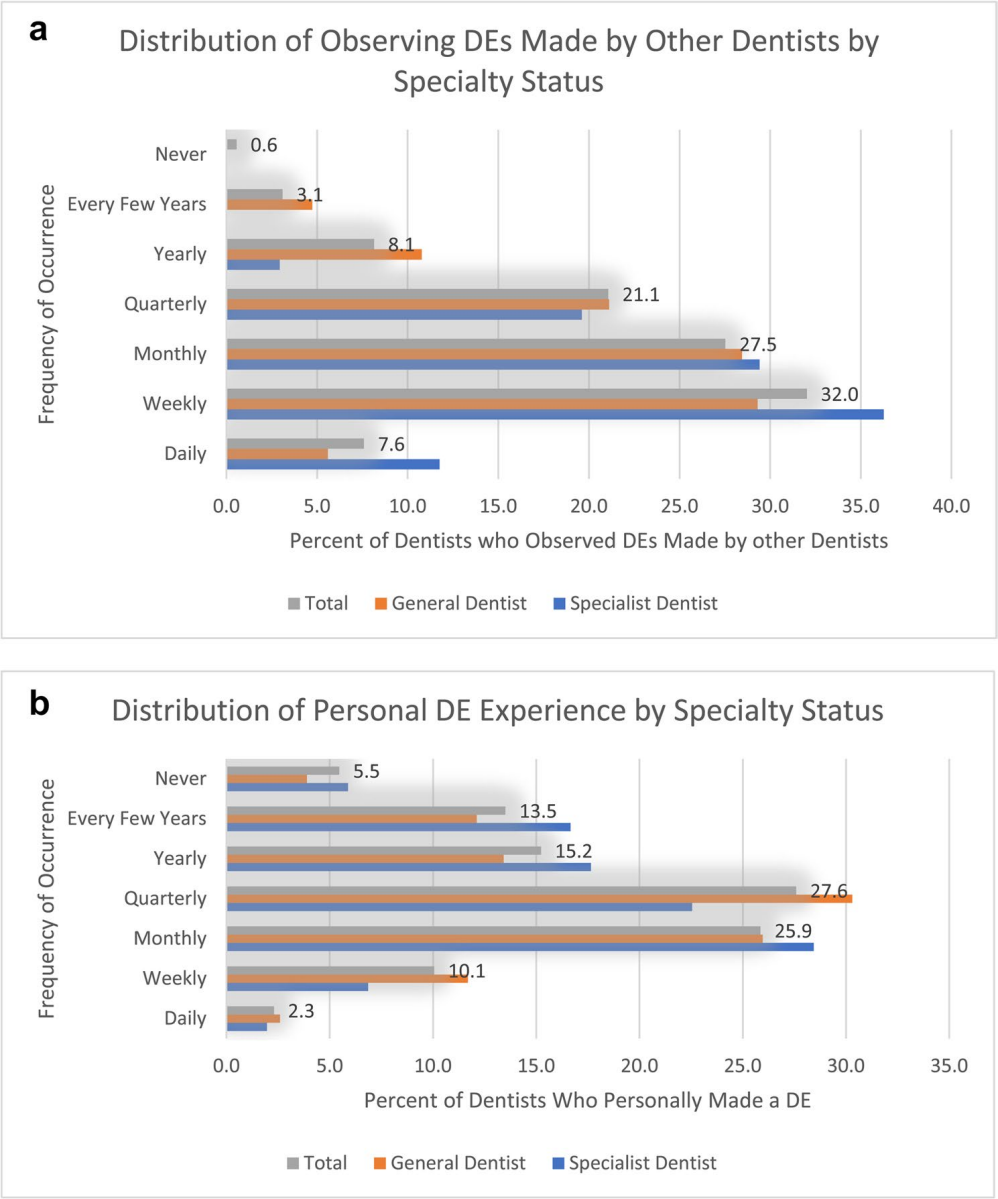
**a** Distribution of observing diagnostic errors made by other dentists by specialty. **b** Distribution of personal diagnostic error experience by specialty status

Dentists reported observing DEs made by other dentists more frequently than they reported personally experiencing/making such errors. About 40% of dentists reported observing a DE made by another dentist on a daily or weekly basis, while only 12.4% admitted to personally making a DE with the same frequency (Fig. 1a and b). Similarly, 5.5% of dentists reported never making a DE personally, but only 0.6% had never observed another dentist making a DE.

### Dental conditions frequently associated with DEs

**Fig. 2:**
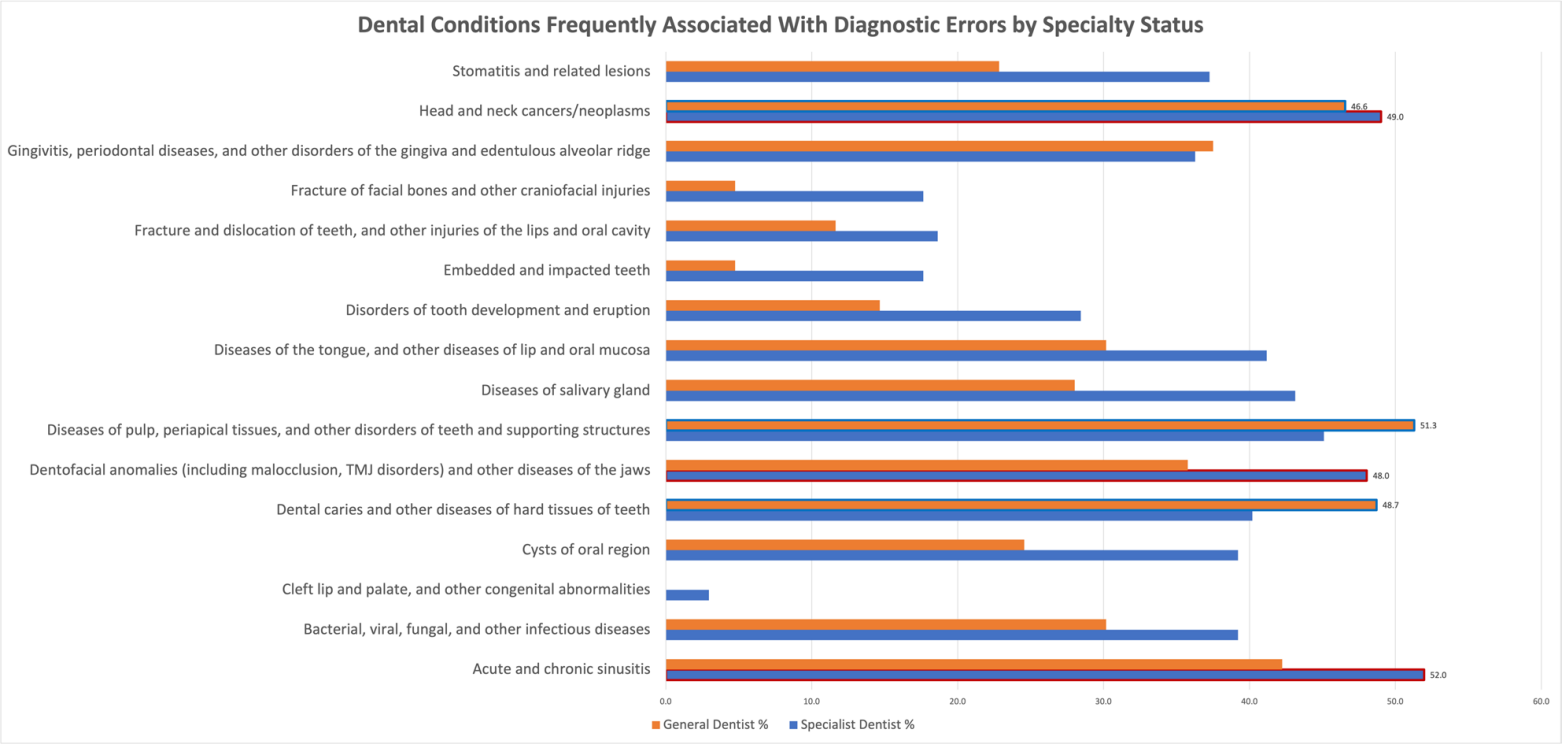
Dental conditions most frequently associated with diagnostic errors stratified by specialty status

Respondents most frequently selected these three dental conditions as associated with DEs: diseases of pulp, periapical tissues, and other disorders of the teeth and supporting structures (45%), acute and chronic sinusitis (44.6%), and head and neck cancers/neoplasms (43.9%). When participants were stratified by general vs. specialist dentists, specialists predominantly selected acute and chronic sinusitis (52%), head and neck cancers/neoplasms (49%), and dentofacial anomalies and other diseases of the jaw (48%), as the dental conditions commonly associated with DEs. Conversely, general dentists selected diseases of pulp/periapical tissues and other disorders of teeth and supporting structures (51.3%), dental caries and other diseases of hard tissues of teeth (48.7%), and head and neck cancers/neoplasms (46.6%), as the dental conditions commonly associated with DEs (Fig. 2

Other dental conditions that were frequently selected by both general and specialist dentists included: gingivitis, periodontal diseases, and other disorders of the gingiva and edentulous alveolar ridge (34.4%); bacterial, viral, fungal, and other infectious diseases (33.1%); and diseases of the salivary gland (31.8%).

### DE failure points and contributory factors

Approximately half of the dentists surveyed identified testing (chairside, pathology, radiology) (52.5%) and the assessment (51.8%) phases as the most frequent failure points in the diagnostic process that were associated with the occurrence of a DE (Table 2).

**Table 2:** DEER stages of the diagnostic process frequently associated with diagnostic errors

DEER Diagnostic Process Stages (*n* = 400)	*n*	%
Access / Presentation	195	48.8
History	200	50.0
Physical Exam	185	46.3
Tests (Chairside/Pathology/Radiology)	210	52.5
Assessment	207	51.8
Referral / Consultation	153	38.3
Monitoring / Follow-up	158	39.5

Respondents were allowed to select all options that applied

^*^DEER= Diagnostic Error Evaluation and Research

Among cognitive-related contributory factors, incomplete medical and dental history-taking and oral examination (63.6%) and missing a noticeable oral disease sign or symptom (61%) were most frequently selected. Poor communication between providers and patients (57.7%), the inexperience of dental staff (48.9%), and the unavailability of relevant diagnostic resources or equipment (e.g. CT, panoramic X-ray) (47.1%), were the most frequently selected system-related contributory factors. For situational factors, providers most frequently selected overconfidence about one’s own diagnostic ability (60.2%) and having an excessive workload or unrealistic billing or patient volume targets (56.2%), as the most common contributory factors (Table 3).

**Table 3:** Cognitive, systems-related, and situational contributory factors for diagnostic errors

	*n*	%
Cognitive Factors (*n* = 385)		
Incomplete history taking or examination	245	63.6
Failure to consider other possibilities once an initial diagnosis has been reached	225	58.4
Over- or underestimating the meaningfulness of a clinical finding	209	54.3
Drawing an inappropriate conclusion from the available data	203	52.7
Missing a symptom or sign that should be noticeable	235	61.0
System-Related Factors (*n* = 378)		
Inadequate staffing levels	54	14.3
Inexperience of dental staff	185	48.9
Poor communication	218	57.7
Unavailability of resources/equipment (e.g. CT scan, panoramic X-ray)	178	47.1
Technical problems (e.g. equipment not working correctly)	62	16.4
Lack of insurance coverage for additional diagnostic tests	157	41.5
Inadequate information systems (e.g. electronic patient records, diagnostic codes)	56	14.8
Lack of a mandatory requirement to document diagnoses or use diagnostic codes	87	23.0
Situational Factors (*n* = 372)		
Excessive workload or unrealistic clinical targets	209	56.2
Provider fatigue	174	46.8
Being misled by advice or anticipated advice from other providers	95	25.5
Overconfidence about one’s own diagnostic ability	224	60.2
Having an attitude towards the patient either of dislike or of fondness	82	22.0

Respondents were allowed to select all options that applied

### Diagnostic errors education and training

Only a third (32.8%), of older graduates (who graduated 11 or more years ago) reported receiving training in DE during their predoctoral education compared to over half (54.7%) of recent graduates (who graduated within 10 years or less), who received predoctoral DE training. Notably, about half of all respondents received neither predoctoral or postdoctoral formal DE training (45.5%). Furthermore, when stratified by specialty status, more general dentists reported receiving no formal DE training, while more specialists received it at the postdoctoral level.

### Clinician and system-focused interventions

The most efficacious clinician-focused interventions included increased training in diagnostic reasoning skills (i.e. predoctoral or postdoctoral programs, continuing education) (37.2%) and increasing the time spent in clinical encounters with patients (19.8%). The most frequently recommended system-focused interventions included increased availability or access to specialists (31.2%) and establishing a non-punitive feedback system to learn from errors (23.3%) (Table 4).

**Table 4:** Clinician and system-focused interventions most effective at preventing or reducing diagnostic errors

	*n*	%
Clinician-Focused Interventions (*n* = 368)		
Asking for a second opinion	63	17.1
Close follow-up of test results or patient symptoms to ensure that the diagnosis is correct	50	13.6
Increasing the time spent in clinical encounters with patients	73	19.8
Improving teamwork and communication within the health care team	27	7.3
Increasing training in diagnostic reasoning skills (i.e. predoctoral or postdoctoral programs, continuing education)	137	37.2
Increasing awareness about diagnostic uncertainty among patients and families	18	4.9
System-Focused Interventions (*n* = 365)		
Increased access to and availability of specialists	114	31.2
Increased access to diagnostic tools and equipment within the dental office	64	17.5
Widespread use of EHR	13	3.6
Widespread availability of diagnostic codes or terminology	6	1.6
A mandatory requirement to document diagnostic codes in the EHR or for billing	11	3.0
Establishing a non-punitive feedback system to learn from errors	85	23.3
Improved feedback pathways to communicate changes in diagnosis	34	9.3
Improved access to electronic diagnostic decision support tools and reference texts	38	10.4

Respondents were allowed to select only one option

^*^EHR= electronic health records

**Fig. 3:**
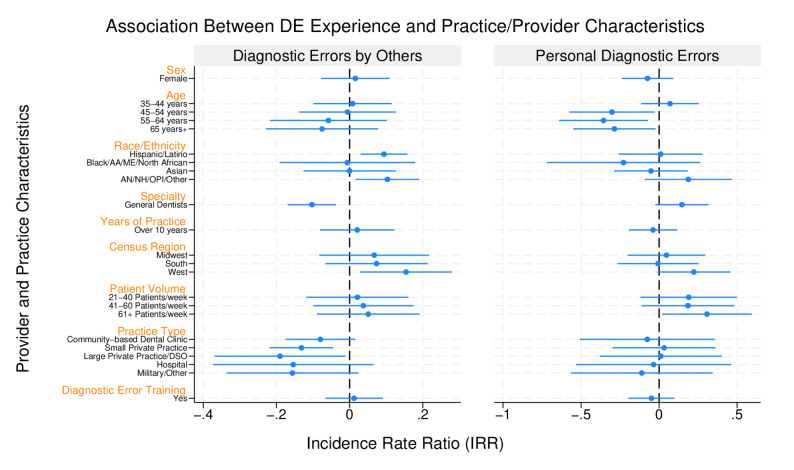
Association between diagnostic error experience and practice/provider characteristics

Using a generalized linear model, we assessed the association between the frequency of observing a DE or personally making a DE, with provider and practice characteristics. *Observing DEs Made by Other Dentists*: General dentists (IRR:0.90; 95% CI: 0.84–0.96; *p* < 0.01) were less likely to observe DEs made by other dentists compared to specialists. Dentists who worked in small/solo private practices (centers (IRR: 0.88; 95% CI: 0.80–0.96, *p* < 0.01), and large private practices (IRR: 0.83; 95% CI: 0.69–0.99; *p* = 0.04) were also significantly less likely to notice DEs made by others when compared to providers who worked for academic dental. Compared to dentists who practiced in the Northeast, dentists in the West (IRR: 1.17; 95%CI: 1.03–1.32; *p* = 0.02) had a higher likelihood of observing DEs made by other dentists. Providers who identified as Hispanic or Latino (IRR:1.10; 95%CI: 1.03–1.17; *p* < 0.01), AI/NH/AN, OPI, or Other (IRR: 1.11; 95%CI: 1.02–1.21; *p* = 0.02) were significantly more likely to notice DEs made by other dentists compared to those who identified as non-Hispanic Whites. There were no significant associations between the frequency of observing DEs made by other providers and the dentists’ age, gender, or patient volume (Fig. 3).

*Personal DE Experience*: Dentists who were aged over 45 years (45–54, 55–64, and 65+) (IRR: 0.99, 0.94, 0.93; 95% CI: 0.87–1.13, 0.80–1.11, 0.80–1.08; *p* = 0.03, 0.01, 0.03 respectively), were significantly less likely to report personally making a DE compared to their younger colleagues (18–34 years). Furthermore, dentists who attended to 61 + patients per week were significantly more likely to report making a DE than providers who saw fewer patients (1–20 patients per week) (IRR: 1.36; 95% CI: 1.02–1.81; *p* = 0.04) (Fig. 3). There were no significant associations observed between the frequency of dentists reporting personally making a DE and the dentists’ gender, race/ethnicity, specialty, practice location, or practice setting (Supplement 2).

## Discussion

This study provides a quantitative analysis of US dentists’ perspectives of DEs. It specifically assesses the frequency of DE experiences, contributory factors, potential interventions, and their associations with provider and practice characteristics. It offers a foundational roadmap for future efforts to mitigate DEs in dentistry [21, 23, 36]. 

Although the literature on DEs in dentistry remains limited [11, 12, 20, 21, 37, 38], our study findings align with prior research in dentistry. This study revealed that DEs occur frequently in dentistry. 38.3% of dentists reported personally making a DE, while 67.1% of dentists reported observing others making a DE at least monthly. These rates are notably high and warrant further investigation. When compared to an electronic dental record (EDR) review of periodontal disease diagnosis, this rate is higher than the 32% misclassified diagnoses observed among predoctoral dental students, or the 29% observed among dental faculty [11]. Some potential explanations could be the variations in clinical complexity, clinical documentation standards, sensitivity and specificity of diagnostic tools, poor regulatory oversight, and the absence of reporting requirements for treatment outcomes. This implies that most dentists are unaware of their own diagnostic performance unless a patient complains or files a malpractice lawsuit. One solution could be the periodic review of diagnostic performance at the provider and practice levels, and the use of a mandatory reporting system. Dental electronic record companies also need to standardize the reporting of quality measures through quality dashboards as part of routine practice insights, that showcase each provider’s performance with their patient population [39]. 

It was unsurprising that the most frequently selected dental conditions by dentists were: diseases of the pulp, periapical tissues, and other disorders of the teeth and supporting structures, acute and chronic sinusitis, and head and neck cancers/neoplasms. The first two are frequently encountered by most general dentists. It has also been documented in the literature that general dentists exhibit sub-optimal diagnostic performance with oral cancers and may often delay referrals for suspicious lesions/swellings [40–44]. In one study from the United Kingdom (UK), 14% of reviewed oral cancer cases were delayed beyond the recommended two-week period [45]. Better education is needed for dentists to recognize early signs and symptoms that precede oral cancer diagnoses such as, unhealed tooth extraction socket [45, 46]. 

In this study, dentists most frequently selected testing and assessment as the phases of the diagnostic process where breakdowns could occur leading to DEs. This aligns with a study of internal medicine physicians in the US that had similar observations [34]. Specifically, the importance of ordering appropriate tests and radiographs, proper patient positioning and test/radiographic interpretation, as well as the prompt follow up of abnormal findings, in arriving at accurate diagnoses, needs to be emphasized. Further research is needed to understand how each of these factors impact the diagnostic performance of dentists. Clinical decision support tools that aid in the flagging of abnormal test results, annotation of radiographs, and/or embedding of clinical guidelines, can be helpful aids for clinicians to minimize these errors [47, 48]. 

Of the systems-related DE contributory factors, poor communication emerged as the most frequently selected factor by dentists, aligning with communication and teamwork challenges observed in internal medicine [1]. This study also revealed that ‘incomplete oral examination’ and ‘medical and dental history-taking’ were the primary failure points associated with cognitive-related contributory factors to dental DEs, which is similar to the observation in the study of pediatricians [33]. Participating dentists also frequently selected ‘excessive workload’ as the primary situational factor that contributes to DEs. Furthermore, dentists who attended to 61 + patients per week were more likely to report personally making a DE. These results mirror similar observations among dentists which found that diagnostic performance reduced when dentists were under time pressure [49]. Moreover, overconfidence in one’s diagnostic abilities was reported by more than half of dentists, echoing known trends of physician overconfidence in medicine [50, 51]. 

This study revealed that specialists were also more likely to notice DEs made by other dentists, possibly due to receiving referrals from complications resulting from DEs (Fig. 3). As future initiatives aim to enhance dentists’ understanding of DEs and implement interventions to reduce their occurrence, it is important to provide DE training that is tailored to both general dentists and dental specialists. Furthermore, the use of technology, such as artificial intelligence (AI) as diagnostic aids, teledentistry, clinical decision support tools, could foster the diagnostic performance of general dentists in the absence/shortage of specialists, and promote better access to dental care [52–61]. 

This study also unveiled an evolving landscape in DE education, demonstrating subtle improvements over time. Nearly half of recent dental graduates received predoctoral DE training, contrasting with only a third of graduates from earlier cohorts. While this trend indicates an increasing integration of DE education within dental school curricula, it also underscores the need for universal adoption of DE education across all dental schools given the fact that nearly half of dentists reported no formal education on diagnostic errors. Integrating DE training into continuing education programs, as well as dental school and residency curricula, presents a promising resolution for reducing DE occurrence and ultimately improving patient safety.

One avenue to enhance DE education within dentistry could involve emulating practices observed in the medical field, notably through the implementation of morbidity and mortality (M&M) rounds [62]. These sessions, conducted regularly in numerous medical specialties, serve as platforms for the comprehensive review and discussion of complex patient cases with the overarching objectives of reducing medical errors, enhancing interprofessional communication, and providing specialized education within the respective medical disciplines [63]. Although a rare occurrence in dentistry, one dental university hospital in France embraced M&M sessions, and reported significant enhancements in trainee learning and performance outcomes after implementation [64]. Broader adoption of M&M rounds in dentistry, or analogous methodologies facilitating nonjudgmental examination and analysis of complex patient cases, e.g., virtual M&Ms through dental practice-based research networks (DPBRN) or communities of practice hold the potential for significantly reducing DEs. In addition, incorporating systematic approaches, such as checklists and provider-automated decision support, could further bolster improvements in diagnostic performance [65–67]. 

This study had several limitations. First, data collection relied on the integrity and transparency of providers, without verification through electronic medical records or patient corroboration. However, dentistry is notorious for the disparate use of diagnostic codes, making electronic record review impractical. Furthermore, we could not rule out the presence of a social desirability bias, stemming from dentists’ concerns regarding malpractice and potential legal repercussions. However, by asking about their observations of other dentists, we sought to tease out these differences. Third, the low response rate suggests that the respondents might have a special interest in the topic. This limits the generalizability of the results. Future studies should seek to further understand the incidence of DEs from providers by stratifying each specialty, using AI or natural language processing of electronic dental records, and testing some of the recommended strategies for reducing them.

## Conclusion

Dental practitioners offer valuable insight into the occurrence of DE, identifying several contributory factors and suggesting pathways for error prevention. Incomplete history-taking and examination, poor communication, and excessive workload were among the primary contributory factors most frequently selected by dentists, suggesting the need for a multifaceted approach to addressing DEs. Study participants identified several potential interventions to help mitigate DE occurrences, such as providing adequate DE education to students and postgraduate trainees, increasing access to specialists, and establishing non-punitive feedback systems. As the field of dentistry endeavors to reduce DEs, dentists need to embrace the development and implementation of preventive strategies that can improve diagnostic performance.

## Supplementary Information

Supplementary Material 1.

Supplementary Material 2.

Supplementary Material 3.

Supplementary Material 4.

## Data Availability

The datasets used and/or analyzed during the current study are available from the corresponding author upon reasonable request.

## Abbreviations

DE: Diagnostic Error
US: United States
ADA: American Dental Association
AI/AN/NH/OPI: American Indian; Alaskan Native; Native Hawaiian; Other Pacific Islander
DEER: Diagnostic Error Evaluation and Research
CT: Computed Tomography
EHR: Electronic Health Records
CI: Confidence interval
FQHC: Federally Qualified Health Center
IHS: Indian Health Service
DSO: Dental Service Organization
IRR: Incidence Rate Ratio
TMJ: Temporomandibular joint
AI: Artificial Intelligence
M&M: Morbidity and Mortality
DPBRN: Dental Practice-based Research Network
