# Supplementary material for: Unveiling dental diagnostic dilemmas: a national survey of US dentists
Source: BMC Oral Health. 2025 Dec 23;26:172. doi: 10.1186/s12903-025-07531-9 (PMC12836924; doi:10.1186/s12903-025-07531-9)
Supplement: Supplementary file 1 — Supplementary Material 1. [file 12903_2025_7531_MOESM1_ESM.docx]

**Supplemental 2.** Association Between Frequency of Diagnostic Errors and Provider and Practice Characteristics

| Provider/Practice Characteristics | *Observed DEs made by other dentists* | | | *Reported personally making a DE* | | |
| --- | --- | --- | --- | --- | --- | --- |
|  | **IRR** | **95% CI** | **p-value** | **IRR** | **95% CI** | **p-value** |
| Sex |  |  |  |  |  |  |
| Male | **-** |  |  | **-** |  |  |
| Female | 1.02 | 0.93-0.1.12 | 0.74 | 0.93 | 0.79-1.10 | 0.38 |
|  |  |  |  |  |  |  |
| Age (years) |  |  |  |  |  |  |
| 18-34 | - |  |  | - |  |  |
| 35-44 | 1.01 | 0.91-1.12 | 0.88 | 1.07 | 0.89-1.29 | 0.46 |
| 45-54 | 0.99 | 0.87-1.13 | 0.93 | 0.74 | 0.56-0.97 | **0.03*** |
| 55-64 | 0.94 | 0.80-1.11 | 0.48 | 0.70 | 0.53-0.93 | **0.01*** |
| 65+ | 0.93 | 0.80-1.08 | 0.34 | 0.75 | 0.58-0.98 | **0.03*** |
|  |  |  |  |  |  |  |
| Race/Ethnicity |  |  |  |  |  |  |
| White (non-Hispanic) | - |  |  | - |  |  |
| Hispanic or Latino | 1.10 | 1.03-1.17 | **0.004*** | 1.01 | 0.77-1.32 | 0.95 |
| Black/AA/ME/NA | 0.99 | 0.83-1.20 | 0.95 | 0.80 | 0.49-1.30 | 0.36 |
| Asian | 1.00 | 0.88-1.13 | 1.00 | 0.95 | 0.75-1.20 | 0.67 |
| AN/NH/OPI/ Other | 1.11 | 1.02-1.21 | **0.02*** | 1.21 | 0.91-1.59 | 0.19 |
|  |  |  |  |  |  |  |
| Specialty |  |  |  |  |  |  |
| Specialist Dentists | - |  |  | - |  |  |
| General Dentists | 0.90 | 0.84-0.96 | **0.002*** | 1.16 | 0.98-1.37 | 0.09 |
|  |  |  |  |  |  |  |
| Years in Practice |  |  |  |  |  |  |
| 0-10 years | - |  |  | - |  |  |
| 11 years+ | 1.02 | 0.92-1.13 | 0.69 | 0.96 | 0.82-1.12 | 0.62 |
|  |  |  |  |  |  |  |
| Practice Type |  |  |  |  |  |  |
| Academic Dental Center | - |  |  | - |  |  |
| Community-based dental clinic (e.g. FQHC, FQHC Look-alike, IHS, community health center) | 0.92 | 0.84-1.02 | 0.10 | 0.93 | 0.60-1.43 | 0.73 |
| Small Private Practice (i.e. solo, small group (2-9 dentists)) | 0.88 | 0.80-0.96 | **0.003*** | 1.03 | 0.74-1.43 | 0.85 |
| Large Private Practice (i.e. large group (10+ dentists), dental service organization, managed care organization) | 0.83 | 0.69-0.99 | **0.04*** | 1.01 | 0.68-1.49 | 0.96 |
| Hospital | 0.86 | 0.69-1.07 | 0.17 | 0.97 | 0.59-1.59 | 0.89 |
| Military/Other | 0.86 | 0.71-1.02 | 0.09 | 0.89 | 0.57-1.41 | 0.63 |
|  |  |  |  |  |  |  |
| Patient Volume (patients/week) |  |  |  |  |  |  |
| 1-20 | - |  |  | - |  |  |
| 21-40 | 1.02 | 0.89-1.17 | 0.77 | 1.21 | 0.89-1.65 | 0.23 |
| 41-60 | 1.04 | 0.91-1.19 | 0.59 | 1.20 | 0.89-1.62 | 0.22 |
| 61+ | 1.05 | 0.91-1.21 | 0.48 | 1.36 | 1.02-1.81 | **0.04*** |
|  |  |  |  |  |  |  |
| Census Region  (Practice Location) |  |  |  |  |  |  |
| Northeast | - |  |  | - |  |  |
| Midwest | 1.07 | 0.92-1.24 | 0.38 | 1.05 | 0.82-1.34 | 0.71 |
| South | 1.08 | 0.94-1.24 | 0.94 | 0.99 | 0.77-1.29 | 0.96 |
| West | 1.17 | 1.03-1.32 | **0.02*** | 1.25 | 0.99-1.58 | 0.06 |
| DE Training |  |  |  |  |  |  |
| No | - |  |  | - |  |  |
| Yes | 1.01 | 0.94-1.16 | 0.76 | 0.95 | 0.82-1.10 | 0.51 |
| AA= African American; ME= Middle Eastern; NA= North African; AI= American Indian; AN= Alaskan Native; NH: Native Hawaiian; OPI: Other Pacific Islander; FQHC= Federally Qualified Health Centers; HIS= Indian Health Service | | | | | | |
