# Supplementary material for: Unveiling dental diagnostic dilemmas: a national survey of US dentists
Source: BMC Oral Health. 2025 Dec 23;26:172. doi: 10.1186/s12903-025-07531-9 (PMC12836924; doi:10.1186/s12903-025-07531-9)
Supplement: Supplementary file 2 — Supplementary Material 2. [file 12903_2025_7531_MOESM2_ESM.pdf]

A National Survey of Dental Diagnostic Errors

We are asking you to take part in a study being conducted by researchers at the [redacted] Being in this study is optional.

If you choose to be in the study, please complete this survey. This survey will help us learn more about the common types of diagnostic errors that occur in the dental care setting and their contributory factors.

In the United States (US), an estimated 5% of adults experience diagnostic errors in the outpatient setting. In its 2015 report, Improving Diagnosis in Health Care, the National Academy of Medicine (NAM) stated, "Improving the diagnostic process is … a moral, professional, and public health imperative." To meet this imperative, dentists need to be able to accurately assess their current levels of diagnostic performance, understand the factors that contribute to dental diagnostic failures, and develop innovative strategies to improve the quality of their diagnoses.

The survey will take about 10-15 minutes to complete.

The survey is anonymous, and no one will be able to link your answers back to you. Please do not include your name or other information that could be used to identify you in the survey responses.

Questions? Please contact the research team at [redacted] If you have questions or concerns about your rights as a research participant, you can call the [redacted]

Section 1: Professional Opinion About Diagnostic Errors in Dentistry

In this section, we would like to learn about your professional opinion of diagnostic errors in dentistry.

Section 1: Professional Opinion About Diagnostic Errors in Dentistry

A diagnostic error is defined as a diagnosis that was unintentionally delayed, wrong, or missed as judged from the eventual appreciation of more definitive information.

Delayed (i.e. sufficient information was available earlier) Wrong (i.e. another diagnosis was made before the correct one) Missed (i.e. no diagnosis was ever made)

Please select the dental conditions that are most frequently prone to diagnostic errors (Select all that apply)

☐ Acute and chronic sinusitis
☐ Bacterial, viral, fungal, and other infectious diseases
☐ Cleft lip and palate, and other congenital abnormalities
☐ Cysts of oral region
☐ Dental caries and other diseases of hard tissues of teeth
☐ Dentofacial anomalies (including malocclusion, TMJ disorders) and other diseases of the jaws
☐ Diseases of pulp, periapical tissues, and other disorders of teeth and supporting structures
☐ Diseases of salivary gland
☐ Diseases of the tongue, and other diseases of lip and oral mucosa
☐ Disorders of tooth development and eruption
☐ Embedded and impacted teeth
☐ Fracture and dislocation of teeth, and other injuries of the lips and oral cavity
☐ Fracture of facial bones and other craniofacial injuries
☐ Gingivitis, periodontal diseases, and other disorders of the gingiva and edentulous alveolar ridge
☐ Head and neck cancers/neoplasms
☐ Stomatitis and related lesions

Section 1: Professional Opinion About Diagnostic Errors in Dentistry

A diagnostic error is defined as a diagnosis that was unintentionally delayed, wrong, or missed as judged from the eventual appreciation of more definitive information.

Delayed (i.e. sufficient information was available earlier) Wrong (i.e. another diagnosis was made before the correct one) Missed (i.e. no diagnosis was ever made)

Please select the steps in the diagnostic process that most often lead to diagnostic errors (Select all that apply)

- ☐ Access/ Presentation. e.g. Delays with a patient presenting to the dental office or accessing dental care
- ☐ History. e.g. Failures/Delays with eliciting, interpreting, weighing, or following up on a critical piece of history data
- ☐ Physical Exam. e.g. Failures/ Delays in eliciting, interpreting, weighing, or following up on a critical exam finding
- ☐ Tests (Chairside/ Pathology/ Radiology). e.g. Failures/ Delays/Errors with ordering, performing, processing, interpreting, or following up on needed tests, radiographs, or pathology results
- ☐ Assessment. e.g. Failures/ Delays/ Errors in considering, weighing, or prioritizing differential diagnoses, the urgency of symptoms, and/or likelihood of complications
- ☐ Referral/ Consultation. e.g. Failures/ Delays/ Errors in ordering or following up on referrals, or obtaining consultation
- ☐ Monitoring/ Follow-up. e.g. Failures/ Delays with monitoring or following up on a patient's symptoms and recovery.

Section 1: Professional Opinion About Diagnostic Errors in Dentistry

A diagnostic error is defined as a diagnosis that was unintentionally delayed, wrong, or missed as judged from the eventual appreciation of more definitive information.

Delayed (i.e. sufficient information was available earlier) Wrong (i.e. another diagnosis was made before the correct one) Missed (i.e. no diagnosis was ever made)

Please select the cognitive factors that most often contribute to the occurrence of diagnostic errors in dentistry (Select all that apply)

☐ Incomplete history taking or examination
☐ Failure to consider other possibilities once an initial diagnosis has been reached
☐ Over- or under-estimating the meaningfulness of a clinical finding
☐ Drawing an inappropriate conclusion from the available data
☐ Missing a symptom or sign that should be noticeable

Section 1: Professional Opinion About Diagnostic Errors in Dentistry

A diagnostic error is defined as a diagnosis that was unintentionally delayed, wrong, or missed as judged from the eventual appreciation of more definitive information.

Delayed (i.e. sufficient information was available earlier) Wrong (i.e. another diagnosis was made before the correct one) Missed (i.e. no diagnosis was ever made)

Please select the system-related factors that most often contribute to the occurrence of diagnostic errors in dentistry (Select all that apply)

☐ Inadequate staffing levels
☐ Inexperience of dental staff
☐ Poor communication
☐ Unavailability of resources/ equipment (e.g. CT scan, panoramic x-ray)
☐ Technical problems (e.g. equipment not working correctly)
☐ Lack of insurance coverage for additional diagnostic tests
☐ Inadequate information systems (e.g. electronic patient records, diagnostic codes)
☐ Lack of a mandatory requirement to document diagnoses or use diagnostic codes

Section 1: Professional Opinion About Diagnostic Errors in Dentistry

A diagnostic error is defined as a diagnosis that was unintentionally delayed, wrong, or missed as judged from the eventual appreciation of more definitive information.

Delayed (i.e. sufficient information was available earlier) Wrong (i.e. another diagnosis was made before the correct one) Missed (i.e. no diagnosis was ever made)

Please select the situational factors that most often contribute to the occurrence of diagnostic errors in dentistry (Select all that apply)

- ☐ Excessive workload or unrealistic clinical targets
- ☐ Provider fatigue
- ☐ Being misled by advice or anticipated advice from other providers
- ☐ Over-confidence about one's own diagnostic ability
- ☐ Having an attitude towards the patient either of dislike or of fondness

Section 1: Professional Opinion About Diagnostic Errors in Dentistry

A diagnostic error is defined as a diagnosis that was unintentionally delayed, wrong, or missed as judged from the eventual appreciation of more definitive information.

Delayed (i.e. sufficient information was available earlier) Wrong (i.e. another diagnosis was made before the correct one) Missed (i.e. no diagnosis was ever made)

Please select the clinician-focused intervention that will be most effective at preventing or reducing diagnostic errors made by dentists

◯ Asking for a second opinion
◯ Close follow-up of test results or patient symptoms to ensure that the diagnosis is correct
◯ Increasing the time spent in clinical encounters with patients
◯ Improving teamwork and communication within the health care team
◯ Increasing training in diagnostic reasoning skills (i.e. predoctoral or postdoctoral programs, continuing education)
◯ Increasing awareness about diagnostic uncertainty amongst patients and families

Section 1: Professional Opinion About Diagnostic Errors in Dentistry

A diagnostic error is defined as a diagnosis that was unintentionally delayed, wrong, or missed as judged from the eventual appreciation of more definitive information.

Delayed (i.e. sufficient information was available earlier) Wrong (i.e. another diagnosis was made before the correct one) Missed (i.e. no diagnosis was ever made)

Please select the system-focused intervention that will be most effective at preventing or reducing diagnostic errors made by dentists

◯ Increased access to and availability of specialists
◯ Increased access to diagnostic tools and equipment within the dental office
◯ Widespread use of electronic health records (EHR)
◯ Widespread availability of diagnostic codes or terminology
◯ A mandatory requirement to document diagnostic codes in the EHR or for billing
◯ Establishing a non-punitive feedback system to learn from errors
◯ Improved feedback pathways to communicate changes in diagnosis
◯ Improved access to electronic diagnostic decision support tools and reference texts

Section 2: Personal Experience with Diagnostic Errors in Dentistry

In this section, we would like to learn more about your past personal experiences with diagnostic errors.

Section 2: Personal Experience with Diagnostic Errors in Dentistry

A diagnostic error is defined as a diagnosis that was unintentionally delayed, wrong, or missed as judged from the eventual appreciation of more definitive information.

Delayed (i.e. sufficient information was available earlier) Wrong (i.e. another diagnosis was made before the correct one) Missed (i.e. no diagnosis was ever made)

How often do you notice diagnostic errors made by other dentists?

◯ Daily
◯ Weekly
◯ Monthly
◯ Quarterly
◯ Yearly
◯ Every few years
◯ Never

Section 2: Personal Experience with Diagnostic Errors in Dentistry

A diagnostic error is defined as a diagnosis that was unintentionally delayed, wrong, or missed as judged from the eventual appreciation of more definitive information.

Delayed (i.e. sufficient information was available earlier) Wrong (i.e. another diagnosis was made before the correct one) Missed (i.e. no diagnosis was ever made)

Please provide a brief description of a diagnostic error that was made by another dentist and its impact on the patient, if known.

Section 2: Personal Experience with Diagnostic Errors in Dentistry

A diagnostic error is defined as a diagnosis that was unintentionally delayed, wrong, or missed as judged from the eventual appreciation of more definitive information.

Delayed (i.e. sufficient information was available earlier) Wrong (i.e. another diagnosis was made before the correct one) Missed (i.e. no diagnosis was ever made)

How often do you believe that you make diagnostic errors?

◯ Daily
◯ Weekly
◯ Monthly
◯ Quarterly
◯ Yearly
◯ Every few years
◯ Never

Section 2: Personal Experience with Diagnostic Errors in Dentistry

A diagnostic error is defined as a diagnosis that was unintentionally delayed, wrong, or missed as judged from the eventual appreciation of more definitive information.

Delayed (i.e. sufficient information was available earlier) Wrong (i.e. another diagnosis was made before the correct one) Missed (i.e. no diagnosis was ever made)

Please provide a brief description of a diagnostic error that you may have made and its impact on the patient, if known.

Section 3: Provider and Practice Characteristics

In this third section, we would like to learn more about you and your clinical practice.

Section 3: Provider and Practice Characteristics

Age

◯ 18-24 years
◯ 25-34 years
◯ 35-44 years
◯ 45-54 years
◯ 55-64 years
◯ 65-74 years
◯ 75-84 years

Gender (Please select the category that best describes you)

◯ Male
◯ Female
◯ Transgender
◯ Other
◯ Prefer not to answer

Race/Ethnicity (Please select all categories that apply)

☐ Hispanic or Latino
☐ White
☐ Black or African American
☐ Middle Eastern or North African
☐ American Indian or Alaska Native
☐ Asian
☐ Native Hawaiian or Other Pacific Islander
☐ Other Race or Ethnicity

What is your primary dental clinical practice area?

◯ General Dentistry
◯ Dental Anesthesiology
◯ Dental Public Health
◯ Endodontics
◯ Prosthodontics
◯ Pediatric Dentistry
◯ Periodontics
◯ Oral and Maxillofacial Surgery
◯ Oral Pathology
◯ Oral Medicine
◯ Oral and Maxillofacial Radiology
◯ Orthodontics
◯ Orofacial Pain

How long have you been in clinical dental practice?

◯ 0-5 years
◯ 6-10 years
◯ 11-15 years
◯ 16-20 years
◯ 21-25 years
◯ ≥ 26 years

Where is your primary clinical dental practice located?

◯ Alabama
◯ Alaska
◯ Arizona
◯ Arkansas
◯ California
◯ Colorado
◯ Connecticut
◯ Delaware
◯ Florida
◯ Georgia
◯ Hawaii
◯ Idaho
◯ Illinois
◯ Indiana
◯ Iowa
◯ Kansas
◯ Kentucky
◯ Louisiana
◯ Maine
◯ Maryland
◯ Massachusetts
◯ Michigan
◯ Minnesota
◯ Mississippi
◯ Missouri
◯ Montana
◯ Nebraska
◯ Nevada
◯ New Hampshire
◯ New Jersey
◯ New Mexico
◯ New York
◯ North Carolina
◯ North Dakota
◯ Ohio
◯ Oklahoma
◯ Oregon
◯ Pennsylvania
◯ Rhode Island
◯ South Carolina
◯ South Dakota
◯ Tennessee
◯ Texas
◯ Utah
◯ Vermont
◯ Virginia
◯ Washington
◯ West Virginia
◯ Wisconsin
◯ Wyoming
◯ Not Applicable

How would you best describe the primary clinical setting in which you practice?

◯ Academic dental center
◯ Community-based dental clinic (e.g. FQHC, FQHC Look-alike, IHS, community health center)
◯ Small private practice (i.e. solo, small group (2-9 dentists))
◯ Large private practice (i.e. large group (10+ dentists), dental service organization, managed care organization)
◯ Hospital
◯ Military
◯ Other
◯ Not applicable

On average, how many patients do you see at your clinical dental practice every week?

◯ 1-20 patients
◯ 21-40 patients
◯ 41-60 patients
◯ 61+ patients
◯ Not applicable

Did you receive any formal training on diagnostic errors in dentistry? (Select all that apply)

☐ Yes, at the predoctoral level
☐ Yes, at the postdoctoral level
☐ No

Please click 'Submit' to complete the survey.
