# Supplementary material for: Unveiling dental diagnostic dilemmas: a national survey of US dentists
Source: BMC Oral Health. 2025 Dec 23;26:172. doi: 10.1186/s12903-025-07531-9 (PMC12836924; doi:10.1186/s12903-025-07531-9)
Supplement: Supplementary file 4 — Supplementary Material 4. [file 12903_2025_7531_MOESM4_ESM.doc]

**Email Information Sheet**

To:

From:

Subject: Understanding Diagnostic Failures in Dentistry (DDF Study)

Hello,

We are doing a research study about the common types of diagnostic failures that occur in the dental care setting and their contributory factors. In the United States (US), an estimated 5% of adults experience a diagnostic error in the outpatient setting. In its 2015 report, *Improving Diagnosis in Health Care,* the National Academy of Medicine (NAM) stated, “Improving the diagnostic process is … a moral, professional, and public health imperative.” In order to meet this imperative, dentists need to be able to accurately assess their current levels of diagnostic performance, understand the factors that contribute to dental diagnostic failures, and develop innovative strategies to improve their quality of diagnoses.

As a practicing dentist, we would like you to participate in this study.

If you choose to be in the study, you will complete an online survey. It will take about 10-15 minutes to complete.

You can skip questions that you do not want to answer or stop the survey at any time. The survey is anonymous, and no one will be able to link your answers back to you. Please do not include your name or other information that could be used to identify you in the survey responses.

Please contact Dr. Eni Obadan-Udoh at [DDFstudy@ucsf.edu](mailto:DDFstudy@ucsf.edu) with questions about this study. If you have questions or concerns about your rights as a research participant, you can call the UCSF Institutional Review Board at 415-476-1814.

Being in this study is optional. If you want to participate, click this link to start the survey:

[*insert link*]

Thank you,

Eni Obadan-Udoh, DDS, MPH, Dr. Med. Sc. 
Assistant Professor,

Director, Dental Public Health Postgraduate Program

Division of Oral Epidemiology and Dental Public Health

Department of Preventive and Restorative Dental Sciences
UCSF School of Dentistry
